# Female Forensic Patients May Be an Atypical Sub-type of Females Presenting Aggressive and Antisocial Behavior

**DOI:** 10.3389/fpsyt.2022.809901

**Published:** 2022-02-10

**Authors:** Sheilagh Hodgins

**Affiliations:** ^1^Département de Psychiatrie et Addictologie, Université de Montréal, et Centre de recherche de l'Institut universitaire en santé mentale de Montréal, Montreal, QC, Canada; ^2^Haina Forensic Psychiatric Institute, Haina, Germany

**Keywords:** antisocial and aggressive behavior, schizophrenia, borderline personality disorders, female, forensic psychiatry

## Abstract

The percentage of forensic psychiatric patients who are female varies from 5 to 13% in Europe, rises to 18% in England and Wales, and sits at 15% in Canada. Similarly, many fewer women than men are incarcerated in correctional facilities. While these statistics supposedly reflect less antisocial and aggressive behavior (AAB) among females than males, not all findings support this supposition. Data from prospective longitudinal studies show that aggressive and antisocial behavior onsets in childhood, and in a small group of females it remains stable across the life-span. Unlike similar males, few of these females are convicted of crimes. This article begins with a review of descriptive studies of females sentenced by criminal courts to treatment in forensic psychiatric hospitals and moves on to present evidence showing that most female AAB does not lead to criminal prosecution. Next, studies of female AAB are reviewed, noting that it onsets in early childhood and, that in a small group remains stable across the life-span. Subsequent sections of the article focus on the two most common mental disorders presented by female forensic patients, schizophrenia and borderline personality disorder, highlighting what is known about the sub-groups of women with these disorders who present AAB. The article concludes with recommendations for earlier identification by psychiatric services of women presenting mental disorders and AAB, treatments to reduce both the symptoms of their mental disorders and their life-long AAB, and the research that is needed in order to improve the effectiveness of these treatments. The real possibilities of prevention of the development of AAB, and even perhaps aspects of the mental disorders that plague female forensic patients, are described.

## Introduction

In most countries fewer women than men are treated within forensic psychiatric services and fewer are incarcerated in correctional facilities. While supposedly this reflects less antisocial behavior and aggressive behavior (AAB) among women than men, not all findings support this supposition. Diagnostic studies report much higher rates of disorders that include symptoms of AAB among males than females, but this sex difference is not evident in prospective longitudinal studies of birth cohorts (1). For example, in a birth cohort of ~1,000 individuals followed to age 32, 7.5% of females and 10.5% of males presented AAB that onset in childhood and remained stable through three decades of life (2). Female aggressive behavior is primarily indirect, reactive, and occurs within relationships (3, 4).

This article begins with a review of descriptive studies of females sentenced by criminal courts to treatment in forensic psychiatric hospitals and moves on to present evidence showing that most female AAB does not lead to criminal prosecution. Next, studies of female AAB are reviewed, noting that AAB onsets in early childhood and, that in a small group of females it remains stable across the life-span. Subsequent sections of the article focus on the two most common mental disorders presented by female forensic patients, schizophrenia and borderline personality disorder, highlighting what is known about the sub-groups of women with these disorders who present AAB. The article concludes with recommendations for earlier identification by psychiatric services of women presenting AAB and treatments to reduce both the symptoms of their mental disorders and their life-long AAB, and the research that is needed in order to improve the effectiveness of these treatments. The real possibilities of prevention of the development of AAB, and even perhaps aspects of the mental disorders that plague female forensic patients, are described.

## Female Forensic Psychiatric Patients

There are few females within forensic psychiatric services (5, 6) and in correctional facilities. Data reported from Belgium, Germany, Latvia, Italy, Ireland, Poland, Portugal, The Netherlands, England and Wales, Scotland, Slovenia, Spain, Finland, France, Croatia, Macedonia and Lithuania in 2013 reported that the prevalence of forensic inpatient beds varied from 1.4 per 100,00 inhabitants in Spain to 23.9 per 100,000 inhabitants in The Netherlands. The percentages of female patients varied from 5% in Slovenia to 18% in England and Wales (7). From 2002 to 2005, in Canada, ~15% of forensic patients were female (8). In most countries, forensic hospitals treat primarily patients with psychotic disorders, however, The Netherlands has an additional forensic system specifically for patients presenting personality disorders.

A Dutch study (9) described a sample of 275 female forensic patients who were in their mid-thirties at the time of admission. Prior to admission, 54% of the women had been convicted of a criminal offense, with a mean age at first conviction of 25 years and an average of four convictions, and 88% had been previously treated in psychiatric services. Just over three-quarters of the women had experienced maltreatment in childhood, 58% were victimized as adults, and 44% of them both in childhood and adulthood. While 54% of the women had children, in 81% of these cases, the children been taken away by social services prior to admission. At the time of the index offense only two-thirds of the women had a home.

Index offenses included homicide (53.8%), arson (23.1%), other violence (14.1%), property offenses (6.4%), and sexual offenses (2.6%). In 88% of cases, victims were involved with the perpetrator and notably there were 24 cases of maternal filicide, seven cases of physical abuse and four cases of sexual abuse of offspring (see also 4). Primary diagnoses included schizophrenia/psychotic disorder (32.9%), substance use disorder (17.4%), depression (12.9%), and post traumatic stress disorder (8.1%). Secondary diagnoses included borderline personality disorder (BOPD) (60.6%) (with another 21% of patients presenting borderline traits), antisocial personality disorder (ASPD) (15.2%) (with another 24% presenting ASPD traits), and narcissistic personality disorder (2.6%) (with another 9% presenting narcissistic traits). Only three of the women obtained scores of 30 or higher on the Psychopathy Checklist-Revised (PCL-R), 19% obtained scores of 23 or higher (a recommended cut-off score for women). The average PCL-R score was 16.5.

During treatment, one-third of the women engaged in aggressive behavior primarily toward staff, and 45% in self-destructive behavior. They were described by staff as being highly manipulative. The mean duration of treatment was 62 months.

Seventy-eight of these women were followed for a 3-year period following discharge (10) during which 18% died and 12% were re-admitted to psychiatric services. Death was associated negatively with interpersonal (facet 1) scores from the PCL-R. Few recidivated, 14 by 3 years post-discharge and 24 at 11 years post-discharge. FAM total items, HCR-20^v3^, clinical items, and START vulnerability scores showed the highest predictive accuracy. Only six women were convicted of violent crimes in the 3 years following discharge, and 14 during 11 years post-discharge. These latter violent convictions were predicted only by HCR-20^v3^ clinical scores and START vulnerabilities score (10).

Another study (11) examined all forensic psychiatric patients in Sweden in 2010, 15% of whom were women aged, on average, 41 years. Prior to admission, 85% of these women had previously been inpatients in psychiatric services, 51% had records of criminal convictions, and 25% convictions for violence. At the time of admission to forensic services, 39% of the women were homeless. Almost half (48%) of the women were diagnosed with schizophrenia spectrum disorders, 9% with mood disorders, and 17% with personality disorders. Most index offenses included some form of violence; 43% crimes related to life and death (e.g., murder, manslaughter, assault), 28% general dangerous crimes (e.g., arson, threat, violence against staff), and 12% liberty and peace (e.g., trafficking, trespassing).

A study in Ontario, Canada of all forensic admissions from 1987 to 2012 resulting in a disposition of Not Criminally Responsible on account of Mental Disorder reported that 14% (362) were women (12). Prior to this admission to forensic services, 91% of the women had been treated in psychiatric services, 65% as inpatients, and 36% had been convicted of crimes. At the time of the index offense, these women were aged, on average, 39 years, 13% of them were homeless, 42% had not graduated from high school, and 21% were employed. More than half (58%) of the index offenses were described as violent and 19% as serious violence. More than three-quarters (77%) of the women were diagnosed with a psychotic disorder, and 22% with mood disorders. Comorbid substance use disorders were reported among 29% and indications of personality disorders among 27%. Another Canadian study provides a similar picture of female forensic patients (13).

Thus, female forensic psychiatric patients present mental disorders that include dysfunctions of emotion and cognition and low levels of psychosocial functioning, most commonly schizophrenia and BOPD, in addition to long histories of AAB and often, psychopathic traits. Most were treated in general psychiatric services before committing the crime that lead to admission to forensic services.

## Female AAB is Hidden From the Criminal Justice System

Among females presenting AAB, forensic patients are distinguished by having been prosecuted in the criminal justice system. Findings from a prospective longitudinal investigation, suggest that despite a long history of AAB, many women escape prosecution. The Dunedin Multidisciplinary Health and Development Study followed a birth cohort of ~500 males and 500 females for more than 40 years, with repeated assessments of behavior, cognition, mental and physical health. At age 32, the males and females characterized by childhood onset AAB were engaging in serious violence and experiencing significant mental health, physical health, and economic problems. A greater proportion of the males (33%) than the females (3%) had been convicted of violent crimes, but 42% of the women and 10% of the males reported hitting a child. While 11.1% of the women reported engaging in violence toward others, informants reported that 47.1% of them had in fact engaged in such violence. Among the males, 30.6% self-reported engaging in violence, and informants reported that 26.7% had engaged in violence (2). Similarly, in a study of forensic patients in The Netherlands, the women had twice as many police contacts without convictions than the men (14). These findings, and others (3) strongly indicate that neither records of arrests or convictions fully capture AAB, especially among females. Women in forensic and correctional facilities may thereby represent a small, atypical, sub-group of females presenting AAB. Importantly, criminal prosecution is influenced by many factors aside from the accused's behavior. Consequently, studies measuring aggressive behavior are more likely to identify both etiological factors for AAB among females and factors promoting prevention and treatment.

We studied 96 teenage girls who consulted a clinic for substance misuse treatment, and their 89 mothers and 52 fathers (15). Forty-three (44.8%) girls reported engaging in at least one violent act (street fight; carried weapon; beaten someone; hurt someone with a weapon). Almost two-thirds (62.8%) of the violent girls and 34.0% of the non-violent were diagnosed with conduct disorder. Univariate comparisons showed that the violent girls, compared to the non-violent, were four times more likely to have a first degree relative with a substance use disorder, three times more likely to have a substance use disorder, three times more likely to have been abused by their mother, three times more likely to have been abused by peers, and two times more likely to have been sexually abused. The violent girls were characterized by significantly more risk factors than the nonviolent girls. Protective factors for violence included maternal warmth, attachment, and parents' attempted understanding.

We followed the girls who had presented conduct disorder for 5 years. At a mean age of 24 years, very few of them met criteria for Antisocial Personality Disorder (ASPD), although they reported significantly higher rates of aggressive behavior than a matched sample of healthy women. Few had graduated from high school, 59% were unemployed, and 48% had given birth on average 10 years earlier than women in Stockholm. Brain scans showed that the women with a history of childhood conduct disorder, as compared to the healthy women, displayed abnormalities of gray and white matter structures even after adjusting for past and current comorbid disorders and maltreatment (16, 17). Both in adolescence and adulthood, the women with prior conduct disorder showed higher levels of psychopathic traits than the healthy women but lower than scores reported for female offenders. Yet, psychopathic traits were associated with abnormalities of neural white matter structures, and notably, the interpersonal facet (glibness, grandiosity, and manipulation) was associated with a white matter abnormality previously observed in adult male offenders presenting the syndrome of psychopathy (18). Thus, in females, conduct disorder prior to age 15 was associated with aggressive behavior from childhood onwards, low academic achievement, by mid-adolescence psychopathic traits higher than levels reported among healthy women that stayed stable for the next 5 years, unemployment, early child birth, neural abnormalities of both gray and white matter, but not diagnoses of ASPD. Thus, female AAB as previously noted remains hidden from view, even though these girls/women display abnormalities of brain structure and functioning (19), endure physical and sexual victimization, hurt others, and fail to support themselves and their offspring.

## AAB Onsets Very Early in Life

A recent review of aggressive behavior stated: “Men are found to use aggression more than females when studies focus on direct forms of aggression (e.g., physical or verbal aggression) and when the target of the aggression is an individual not known to the perpetrator. Conversely, females are found to use aggression more often than males when studies focus on indirect forms of aggression (e.g., psychological or social aggression) and when the target of the aggression is an individual known to the perpetrator” (20). Robust evidence from prospective, longitudinal investigations of birth and population cohorts conducted in different countries confirm that aggressive behavior is observed during the first year of life, that it increases to about the age of 4 years, then declines. All studies identify one group of individuals who display high levels of aggressive behavior through childhood, adolescence, and adulthood (20). In a representative sample of 1,183 Canadian children 12.5% of boys and 11.0% of girls were reported to present the highest levels of aggressive behavior and of indirect aggression from ages 2 to 8 years. Another 1% of boys and <0.63% girls presented high aggressive behavior without indirect aggression and 0.18% of boys and 1% of girls presented indirect aggression with aggressive behavior (21). These same data showed that from 2 years of age onwards, among those presenting high, stable, aggressive behavior, there were more boys (53.6%) than girls (46.4%). From age 4 years, more girls (57.6%) than boys (42.7%) were on a high trajectory of indirect (relational) aggression. While trajectories of aggressive behavior remained relatively stable through adolescence, there was a large increase in the proportions of teenagers engaging in relational aggression. There is now a large body of evidence regarding bullying by both boys and girls (22). Other studies report that from toddlerhood through adolescence (~5% of boys display high levels of physically aggressive behavior as do 1% of girls (23); for a review see (24). As for all types of aggressive behavior, across adolescence, stable high trajectories of both proactive and reactive aggression have been observed (20).

In the Dunedin study (25), comparisons were made of the males and females who presented conduct problems in childhood and whose AAB persisted across the life-span. At age three, within sex comparisons showed that those who presented early onset, persistent, conduct problems differed from other cohort members by displaying more neurological soft signs, and lower scores on the Bailey Motor Test, and in childhood lower IQ and reading scores. Boys, not girls, characterized by early onset, persistent, conduct problems also presented lower heart rate, uncontrolled temperament, and poor memory. From age 7 to 15 years, both females and males with childhood onset, persistent, conduct problems differed from same sex healthy peers as to three individual factors-low IQ, poor reading achievement, and ADHD symptoms, and seven parent characteristics-low socio-economic status, maltreatment and inconsistent parenting, family conflict, poor maternal mental health, low maternal IQ, and parents' criminality. Thus, individual and family risk factors for early onset conduct problems that remained stable across the life-span were mostly similar in males and females.

Callous-unemotional traits, a key antecedent of psychopathy traits, can be identified by age 2 or 3 years. At even younger ages, precursors of callous-unemotional traits are observed (26–28). Among toddlers, callous-unemotional traits are reduced by warm, positive maternal parenting. If not lowered, these traits provoke harsh parenting in the subsequent years that promotes AAB. A study of a randomly selected population sample in the UK estimated that close to half of both the girls and boys with conduct disorder presented elevated levels of callous-unemotional traits (29). These children were five times more likely than others with conduct disorder to show serious conduct problems 3 years later. Prevalence rates for elevated levels of CU traits have ranged from 10 to 32% in community samples and 21% to 50% in clinic-referred samples of children with conduct problems (30). Thus, while CU is observed by age 2 or 3 years, and predictors of CU by six or seven months of age, CU shows change during childhood, but stability in a small group. By adolescence, levels of psychopathic traits appear to be relatively consolidated in both males and females (31).

In a study of a large UK population sample of 9,462 twins, trajectory analyses of callous-unemotional traits rated by teachers at age 7, 9, and 12 years found that 3.4% of the children presented high stable callous-unemotional traits. Nineteen percent of these children were girls. The sub-group displaying high and stable callous-unemotional traits showed the highest levels of conduct problems prior to school entry and by early adolescence their families were described as chaotic, and their parents were using negative discipline (32). The moderate stability and resistance to change of callous-unemotional traits from age 7 to 17 was shown in a study of boys. Only eight of 65 family and individual factors that were examined modified stability of callous-unemotional traits, but only among boys with low, not high, psychopathy scores at age 13 (33).

The stability of conduct problems and callous-unemotional traits is further shown by a US study in which a large sample of US children, at age three, were divided into four groups: 1. no conduct problems, no callous-unemotional traits, no internalizing symptoms; 2. conduct problems alone; 3. conduct problems with callous-unemotional traits; and 4. conduct problems with callous-unemotional traits and internalizing problems. Membership in these groups remained stable up to age 15 (34). This study found no sex differences in the proportions of girls in the four groups. By contrast, other studies of children suggest that more girls than boys present conduct problems, callous-unemotional traits and anxiety, while studies of adolescents suggest that the group with conduct problems and callous-unemotional traits and low anxiety includes few females (34).

Children presenting conduct problems together with callous-unemotional traits and anxiety show heightened threat perception, and greater autonomic and central nervous system reactivity that triggers reactive aggression (35), and have more often experienced maltreatment than children presenting conduct problems and callous-unemotional traits but not anxiety (35–37). Further, changes in autonomic system reactivity following maltreatment vary as a function of callous-unemotional traits (35). Children presenting conduct problems, callous-unemotional traits, and anxiety are fearful, hypersensitive to threat (38), obtain lower than average intelligence scores, do poorly at school, show weak self-regulation (34), and present no deficits in recognizing or responding to emotional expressions (38, 39), despite being more behaviourally and emotionally dysregulated (38).

Importantly, conduct problems and callous-unemotional traits in young girls predict criminality. In a prospective study of 1,241 girls, we found that those rated by their teachers at age 6 as showing conduct problems and callous-unemotional traits were six times more likely than girls without such ratings to be convicted of a non-violent crime by age 24, and those rated by their teachers as presenting callous-unemotional traits with or without conduct problems at age 10 were four times more likely to be convicted of non-violent crimes by age 24 (40).

Taken together, this evidence suggests that women sentenced to forensic psychiatric services may have a life-long history of AAB and the callous-unemotional traits of psychopathy. They have endured physical and sexual maltreatment, done poorly at school and in the job market, had multiple intimate relationships often with men presenting AAB and at a young age given birth to children who are at risk for AAB from a young age. The AAB may often have been in response to real or perceived threat or to curry favor with friends or to achieve some other goal. Treatments and management strategies aimed at reducing AAB by these women are, in fact, tackling a life-long pattern of behavior and personality.

## Mental Disorders Presented by Female Forensic Psychiatric Patients

### Schizophrenia

While studies of forensic psychiatric samples show that most risk factors for violence are similar in males and females, for example similar prevalence of childhood conduct problems (41), clinical factors are more strongly linked to aggressive behavior in females (3, 4). Yet, studies of female forensic patients have not generally focused on aspects of their mental disorders that may be linked to AAB. Schizophrenia is a potent risk factor for violent offending among both females and males (41). While fewer women than men with schizophrenia commit crimes, schizophrenia increases the risk of violent offending to a greater extent among females than males. For example, we examined a birth cohort composed of all the 358,180 persons born in Denmark from 1944 through 1947 followed until they were in their mid-forties. Official criminal records indicated that the risk of a violent crime was elevated 23.2 (14.4–37.4) times among the women with schizophrenia treated in psychiatric services as compared to women never admitted to a psychiatric ward, a much greater increase than that of five found among males (42). These findings suggest that schizophrenia confers a very elevated risk for violence in females. In other words, very few females are convicted of a violent crime, but among those few who develop schizophrenia a much larger proportion are convicted of a violent crime. However, the etiology of AAB among women with schizophrenia may be the same as it is for men with schizophrenia.

As presented in Table 1, comparisons of criminal convictions of women hospitalized in general psychiatry with diagnoses of severe mental illness (primarily schizophrenia) and general population samples of women in the UK, Sweden, and Denmark, again shows that the increase in risk conferred by illness is much greater for females than males. Severe mental illness was associated with a 17 fold increase in risk of a conviction for a violent crime in the UK, 11 fold in Sweden, and 6 fold in Denmark (43). But as noted, many incidents of aggressive behavior, particularly among women, do not lead to criminal prosecution. For example, using the definitions of aggressive behavior and serious violence from the MacArthur study of violence (44), we examined aggressive behavior of females in three different studies that were similar as to age and that had used the same instrument to collect information on aggressive behavior and violence (43). In a UK sample of inpatients with severe mental illness (primarily schizophrenia) 39% had engaged in aggressive behavior and 19% in serious violence (43) in the past 6 months, in a trial of antipsychotic medications that recruited only stable patients with schizophrenia, 21% had engaged in aggressive behavior and 3% violence in the past 6 months (44), and among the patients with schizophrenia in the MacArthur Violence study 44% reported aggressive behavior and 18% violence in the past 10 weeks (43). Even prior to first admission for psychosis, approximately one-third of patients are reported to have engaged in aggressive behavior (45). A meta-analysis reported that male sex was associated with any violence, but there was no sex-difference when examining incidents of serious violence (46). People with schizophrenia are at high risk for physical victimization, more so if they are engaging in aggressive behavior (47). The risk of such victimization varies across countries (48).

**Table 1:** Odds ratios for criminal convictions up to age 30 comparing inpatient samples with severe mental illness to general population samples from three countries.

	**UK inpatient sample compared to UK general population sample^**A**^**	**Swedish inpatient sample compared to Swedish population sample^**B**^**	**Danish inpatient sample compared to Danish population sample^**C**^**
Men			
Conviction for a criminal offense	2.72 (1.90–3.90)	2.15 (1.39–3.33)	2.59 (2.37–2.84)
Conviction for a violent criminal offense	4.86 (3.30–7.16)	4.74 (2.84–7.91)	2.49 (2.10–2.95)
Women			
Conviction for a criminal offense	2.85 (1.63–4.98)	3.78 (2.13–6.69)	3.48 (2.96–4.08)
Conviction for a violent criminal offense	17.24 (8.18–36.32)	11.18 (4.30–29.13)	5.89 (3.60–9.63)

*A. General population statistics available from Chapter 3 of Home Office's ‘Criminal careers of those born between 1953 and 1978' document, available from http://www.homeoffice.gov.uk/rds/pdfs/hosb401.pdf*.

*B. Hodgins S. Mental disorder, intellectual deficiency, and crime. Evidence from a birth cohort. Arch Gen Psychiatry (1992) 49:476–83. doi: https://doi.org/10.1001/archpsyc.1992.01820060056009*.

*C. Hodgins S, Mednick SA, Brennan PA, Schulsinger F, Engberg M. Mental disorder and crime. Evidence from a Danish birth cohort. Arch Gen Psychiatry (1996) 53:489–96. doi: https://doi.org/10.1001/archpsyc.1996.01830060031004*.

*Odds ratios were calculated from data as article presents relative risk ratios*.

*Table from: (3)*.

Thus, while fewer women than men, with schizophrenia, are convicted of crimes and engage in aggressive behavior toward others, schizophrenia confers a greater risk for offending and for aggressive behavior among women than among men. This finding may suggest that schizophrenia symptoms and the associated features such as cognitive, emotional, and psychosocial functioning, are more related to aggressive behavior in women than men. Further, this finding implies that the effective treatment of all aspects of the illness is needed in order to reduce aggressive behavior.

People who develop schizophrenia present multiple difficulties from early childhood onwards. By age two, they show motor abnormalities such as delays in walking and talking and specific neurological soft signs (49), in the subsequent years there is further evidence of motor deficiencies, neurological signs, receptive language deficits (50), lower than average IQ (51), and by mid-childhood psychotic-like-experiences (52). Additionally, a significant minority present conduct disorder. These children are more likely than healthy children to experience maltreatment by adults and by peers (53). Prospectively collected data indicate that 40% of individuals who develop schizophrenia presented conduct problems in childhood (54), while retrospectively collected data from samples of women and men with schizophrenia report that ~20% had a history of conduct problems since childhood. Some estimates among male patients with severe mental illness are as high as 42% presenting childhood onset conduct problems (43). We found that among males, symptoms of conduct disorder were linearly, and positively associated with numbers of convictions for any crime and for violent crimes after controlling for substance misuse (55). In a sample of male and female patients with severe mental illness, we found no sex difference in the link between childhood/adolescent conduct disorder and crime or aggressive behavior after controlling for substance misuse (56).

Females who are developing schizophrenia are hidden among adolescents presenting AAB. For example, we conducted a follow-up study of 1660 males and 332 females who had been treated at the only clinic for adolescent substance misuse in a large urban center in Sweden from 1968 to 1971. When they consulted the clinic, one-third of the females had not used illicit drugs, two-thirds had used alcohol only experimentally or not at all, and 61% had no record of delinquency. Statistics Sweden created a general population sample by randomly selecting for each individual in this clinical sample an individual in the general population with the same sex, month, year, and place of birth. All participants were followed to the age of 50 using national health, criminal, and social service registers. Among the females in the clinical sample compared to those in the general population sample, the risk (expressed as odds ratio) of developing schizophrenia was 8.55 (2.55–28.66) by age 50, much higher than the risk for males 3.79 (2.38–6.05) (57). A similar analysis of younger sample lead to the same results (58).

Thus, by the time a women presenting schizophrenia is admitted to a forensic psychiatric service she typically has a long history of motor, cognitive, and emotional difficulties, and most will also have a history of AAB.

Studies have shown that elevated positive psychotic symptoms during an acute episode, for example at admission to a psychiatric ward, are associated with aggressive behavior in almost all patients. When patients take antipsychotic medication, psychotic symptoms decline as does the aggressive behavior. For example, one study of inpatients included 67 female and 155 male patients with schizophrenia or bipolar disorder. There was no difference in chlorpromazine equivalents of antipsychotic medications taken by the women and men. Thirty-one percent of the women and 43% of the men engaged in physical assaults in the 2 months after admission. Women tended to have a higher total number of physical assaults than men during the initial month after admission, and a faster decrease in assaults during the second month. Although 41% of the males had engaged in aggressive behavior in the community prior to admission, this was true of only 25% of the women (59).

When positive symptoms are lowered by antipsychotic medication, other factors such as an early onset pattern of AAB emerges as the one of the strongest predictors and correlates of AAB (60–62). However, positive psychotic symptoms and aggressive behavior are intricately linked in ways that still elude understanding. A recent randomized controlled trial measured the effects of different antipsychotic medications on positive symptoms and aggressive behavior of men with schizophrenia, half of whom presented a history of AAB since childhood. Among violent patients with schizophrenia, those with conduct disorder prior to age 15, as compared to those without this past disorder, showed greater reductions in aggressive behavior and similar reductions in positive symptoms when taking clozapine as compared to haloperidol. Similarly, a stronger lowering of aggressive behavior was shown among patients with than without prior conduct disorder when taking olanzapine as compared to haloperidol (63). Interestingly, the medication, clozapine, that was most effective in lowering both positive symptoms and aggressive behavior among the men with schizophrenia and a childhood history of AAB, impacts the neurotransmitter serotonin that is strongly linked to aggressive behavior (64). Further, among men with schizophrenia, those with a childhood onset of AAB present distinctive neural differences as compared to those with no childhood history of AAB and some neural characteristics similar to men without schizophrenia with a childhood history of AAB (65). There are no similar studies of females with schizophrenia who present AAB. However, since neither the etiology of schizophrenia (66) nor AAB (67) varies by sex, it is likely that neural abnormalities observed among men presenting schizophrenia and AAB also characterize women with schizophrenia and AAB. More knowledge of the interplay of aspects of schizophrenia and AAB from early childhood onwards is urgently needed.

### Borderline Personality Disorder

As noted above, another disorder commonly diagnosed among female forensic patients is BOPD. The prevalence of BOPD is estimated at 0.7% to 2.3% with most studies reporting a similar prevalence in women and men, but much higher treatment seeking among women (68). Reactive aggressive behavior is recognized as a key feature of BOPD. The aggressive behavior of persons with BOPD is reported to be associated with affective dysregulation, impulsivity, threat hypersensitivity, and empathic functioning and the associated neurobiological abnormalities (69). Women, like men with BOPD, also display traits of psychopathy (70, 71) and/or ASPD (72) that are associated with aggressive behavior. Thus, some of the mechanisms underlying aggressive behavior among women presenting BOPD may be directly related to BOPD, while others are associated with traits of ASPD and psychopathy. The key to understanding the etiology of aggressive behavior among women with BOPD, and to identify effective treatments, risk management strategies, and prevention programs, may be furthering knowledge of the disorder itself.

As with females presenting AAB and those who develop schizophrenia, by the time women with BOPD are admitted to a forensic psychiatry service, they have a long history of difficulties. For example, in a prospective, longitudinal study of 2,232 British twins, at age 12, mothers rated items tapping three core features of adult BOPD: affective instability/dysregulation, impulsivity/behavioural dysregulation, and disturbed relatedness/interpersonal dysfunction. The children who obtained scores at the 95^th^ percentile or higher of the cohort on the borderline personality disorder items were characterized by lower IQs, less well developed theory of mind, low self-control, high levels of impulsivity and externalizing and internalizing problems, and were seven times more likely than the children without these high scores to have experienced maltreatment. They were also more likely than the children without the borderline symptoms to present conduct disorder, depression, anxiety, and psychotic symptoms (73). By age 18, they presented a distinct personality characterized by narrow-mindedness, antagonism, distress, and poor impulse control, and elevated risks for conduct disorder, alcohol use disorder, cannabis use disorder, depression, generalized anxiety disorder, post-traumatic stress disorder, and suicide attempts or self-harm. Not surprisingly given these disorders, they had acquired low educational qualifications, were neither studying or working, smoked, were socially isolated, reported low life satisfaction, had records of criminality, and more physical and sexual maltreatment and crime victimization (74).

Another prospective longitudinal study of 2,450 US adolescent girls assessed clinical, psychosocial, and demographic factors, previously found to be associated with BOPD in late childhood, early and mid-adolescence. Nineteen predictors assessing depressive and anxiety symptoms, self-control, harsh punishment, and poor social and school functioning explained 33.2% of the variance in BOPD symptoms. Factors were identified that distinguished BOPD from conduct disorder and depression (75).

Thus, when women with BOPD are sentenced to forensic psychiatric treatment, they present a long history of emotional dysregulation, conduct problems, lower than average cognitive abilities, failure at school and at work, AAB, and criminality.

## The Role of Maltreatment

As reviewed above, young girls presenting AAB, and those developing schizophrenia or BOPD are at elevated risk for physical and sexual maltreatment by adults and peers. The high risk of victimization persists through adulthood. A Swedish study included 34,903 persons with schizophrenia, 29,692 with bipolar disorder, and a comparison group of 2,763,012 Swedish citizens without these disorders. Six “triggers” of violence were assessed: exposure to violence, parental bereavement, self-harm, traumatic brain injury, unintentional injuries, and substance intoxication. Within individual analyses were conducted to determine whether the “triggers” occurred in the week preceding the commission of a violent crime. The triggers were all associated with an increased risk of violent crime in each group, most strongly among persons with schizophrenia. The trigger most strongly associated with committing a violent crime was violent victimization that increased the risk of violent crime in the following week, 12 times among people with schizophrenia, and eight times among people with bipolar disorder and also among those in the comparison group (76). Aggressive behavior is strongly associated with physical victimization highlighting a vicious circle that likely onsets in childhood and persists across the life-span.

## Conclusion

There are few women in forensic psychiatric hospitals. They suffer from severe mental disorders that are associated with difficulties in multiple domains of functioning accompanied by AAB from early childhood onwards. While most females presenting a life-long history of AAB escape criminal prosecution, the few in forensic psychiatry did not. When they are admitted to general psychiatric services, little, if anything is done to assess, manage, and/or reduce their AAB. Yet, effective treatment at first episode for psychosis could prevent much suffering and harm to others. Knowledge is urgently needed to identify treatments that effectively reduce the symptoms of their mental disorders, increase their level of psychosocial functioning, and reduce their AAB. The research strategy most likely to provide knowledge that would improve the effectiveness of treatment for such women would focus on studies of clinical samples of females with schizophrenia or with BOPD, comparing those with and without AAB. Such studies also have the potential to yield findings about the association of the mental disorder symptoms and AAB.

Current evidence strongly suggests that the factors associated with the mental disorders of women who end up in forensic services are intricately connected with their AAB across developmental stages. The available evidence also shows that the antecedents of these women's mental disorders and AAB emerge very early in life. Aggressive behavior and callous-unemotional traits, motor and language delays, low IQ, emotion dysfunction, and parents presenting AAB and/or mental health problems providing harsh and ineffective parenting are evident prior to school entry such that by age six regular classroom teachers can identify those at elevated risk of adult criminality. Findings from prospective, longitudinal studies already provide a wealth of evidence that could be used to inform childhood prevention programs. Interventions that succeeded in reducing aggressive behavior, impulsivity, and emotion dysregulation in childhood would allow for greater academic success, despite lower than average intelligence, and interventions in early adolescence could prevent substance misuse that would increase the likelihood of completing work training and employment.

Although AAB by females is hidden from view, the consequences for children and others cared for by women, such as the elderly, are substantial and destructive. These women play a critical role in the intergenerational transfer of antisocial behavior as shown by the fact that their offspring present an elevated risk of AAB (77). Females who present AAB disproportionately mate with antisocial males, give birth at a young age, transmit genes that confer vulnerability for antisocial behavior, provide harsh parenting and other adverse rearing conditions to their offspring (77). Thus, preventing the development of women who are treated in forensic psychiatric services will take several generations. Interventions aimed at reducing teen pregnancy would contribute to achieving this objective. Parents who themselves present antisocial and/or aggressive behavior are at increased risk to engage in non-optimal parenting (78), and to physically maltreat their children (24, 78–80). When children are born to young women with histories of AAB, nurse visitation programs can identify mothers who would benefit from parenting programs that reduce conduct problems in their offspring.

Public and mental health policies that allow females to develop such profound disorders and to harm so many victims before sentencing them to forensic care are cruel and wasteful. Science can contribute to prevention and to the reduction of suffering.

## Author Contributions

The author confirms being the sole contributor of this work and has approved it for publication.

## Funding

This work was supported by the Haina Institute of Forensic Psychiatry.

## Conflict of Interest

The author declares that the research was conducted in the absence of any commercial or financial relationships that could be construed as a potential conflict of interest.

## Publisher's Note

All claims expressed in this article are solely those of the authors and do not necessarily represent those of their affiliated organizations, or those of the publisher, the editors and the reviewers. Any product that may be evaluated in this article, or claim that may be made by its manufacturer, is not guaranteed or endorsed by the publisher.
